# Gastroenterology visitation and reminders predict surveillance uptake for patients with adenomas with high-risk features

**DOI:** 10.1038/s41598-021-88376-4

**Published:** 2021-04-22

**Authors:** Anthony Myint, Edgar Corona, Liu Yang, Bao Sean Nguyen, Christina Lin, Marcela Zhou Huang, Paul Shao, Didi Mwengela, Michelle Didero, Ishan Asokan, Alex A. T. Bui, William Hsu, Cleo Maehara, Bita V. Naini, Yuna Kang, Roshan Bastani, Folasade P. May

**Affiliations:** 1 Department of Medicine, Vatche and Tamar Manoukian Division of Digestive Diseases, David Geffen School of Medicine, University of California Los Angeles, Los Angeles, CA USA; 2 Department of Medicine, David Geffen School of Medicine, University of California Los Angeles, Los Angeles, CA USA; 3 Medical and Imaging Informatics, Department of Radiological Sciences, University of California Los Angeles, Los Angeles, CA USA; 4 Department of Pathology, David Geffen School of Medicine, University of California Los Angeles, Los Angeles, CA USA; 5 UCLA Center for Cancer Prevention and Control Research, UCLA Kaiser Permanente Center for Health Equity and Department of Health Policy and Management, Center for Health Science, Fielding School of Public Health and Jonsson Comprehensive Cancer Center, 650 Charles E Young Drive South, Suite A2-125, Los Angeles, CA 90095 USA

**Keywords:** Colorectal cancer, Disease prevention

## Abstract

Individuals diagnosed with colorectal adenomas with high-risk features during screening colonoscopy have increased risk for the development of subsequent adenomas and colorectal cancer. While US guidelines recommend surveillance colonoscopy at 3 years in this high-risk population, surveillance uptake is suboptimal. To inform future interventions to improve surveillance uptake, we sought to assess surveillance rates and identify facilitators of uptake in a large integrated health system. We utilized a cohort of patients with a diagnosis of ≥ 1 tubular adenoma (TA) with high-risk features (TA ≥ 1 cm, TA with villous features, TA with high-grade dysplasia, or ≥ 3 TA of any size) on colonoscopy between 2013 and 2016. Surveillance colonoscopy completion within 3.5 years of diagnosis of an adenoma with high-risk features was our primary outcome. We evaluated surveillance uptake over time and utilized logistic regression to detect factors associated with completion of surveillance colonoscopy. The final cohort was comprised of 405 patients. 172 (42.5%) patients successfully completed surveillance colonoscopy by 3.5 years. Use of a patient reminder (telephone, electronic message, or letter) for due surveillance (adjusted odds = 1.9; 95%CI = 1.2–2.8) and having ≥ 1 gastroenterology (GI) visit after diagnosis of an adenoma with high-risk features (adjusted odds = 2.6; 95%CI = 1.6–4.2) significantly predicted surveillance colonoscopy completion at 3.5 years. For patients diagnosed with adenomas with high-risk features, surveillance colonoscopy uptake is suboptimal and frequently occurs after the 3-year surveillance recommendation. Patient reminders and visitation with GI after index colonoscopy are associated with timely surveillance completion. Our findings highlight potential health system interventions to increase timely surveillance uptake for patients diagnosed with adenomas with high-risk features.

## Introduction

Colorectal cancer (CRC) is the second leading cause of cancer-related mortality in the United States (US)^1^. The classic model of CRC pathogenesis posits that cancer develops in a stepwise fashion from precancerous tubular adenomas (TA) to dysplasia to overt carcinoma. Substantial evidence supports that early detection and removal of precursor lesions through screening and surveillance colonoscopy can reduce subsequent malignancy and cancer-related mortality^2–5^. Colonoscopies performed in individuals without a prior history of colon polyps or CRC are referred to as screening colonoscopies, whereas those performed in individuals with a prior history of colon polyps or CRC are referred to as surveillance colonoscopies. Patients indicated for surveillance colonoscopy can be further stratified for their risk of CRC based on prior adenoma subtype. Historically, the 2006 and 2012 US Multi-Society Task Force (MSTF) guidelines categorized any TA with size ≥ 1 cm, with tubulovillous or villous histology, with high grade dysplasia, or 3 or more TA in the high-risk adenoma (HRA) category and recommended surveillance colonoscopy 3 years after HRA diagnosis^6^. The recently released 2020 MSTF surveillance guidelines continue to recommend 3-year surveillance for TA ≥ 1 cm, adenomas with tubulovillous or villous histology, adenomas with high-grade dysplasia, 5–10 TA, and several additional subgroups for which adherence to surveillance guidelines is important^7^.

Despite evolving CRC surveillance guidelines over time, adherence to surveillance recommendations has been consistently low in the US. In a large retrospective cohort within community practice, only 30.7% of patients diagnosed with advanced tubular adenoma (aTA; defined by having at least one TA ≥ 1 cm, TA with tubulovillous/villous histology, or TA with high-grade dysplasia) and 19.5% of patients with 3 or more non-advanced adenomas completed surveillance by 3 years^8^. In a multisite cohort spanning 4 regional health systems, surveillance uptake at 3.5 years in patients with either 3 or more adenomas and/or 1 or more adenomas with tubulovillous/villous histology ranged between 18.3 and 59.5%^9^.

Despite suboptimal surveillance uptake among individuals with adenomas with high-risk features, we presently lack effective strategies to increase surveillance rates. Ample efforts in public health and clinical research address suboptimal CRC screening rates and evaluate interventions to increase screening among individuals at average-risk for CRC; however, strategies to reduce ongoing risk following diagnosis of an adenoma with high-risk features remain sparse in CRC prevention and control^10^. Non-modifiable predictors for timely surveillance including younger age, having a first-degree relative with CRC, and having ≥ 3 adenomas^8,9,11^, but less is known about modifiable patient-, provider-, and health-system factors that could serve as potential targets for interventions to increase timely surveillance uptake.

Within our health system, the usual process of communicating the need for surveillance colonoscopy for an adenoma with high-risk features generally occurs shortly after the index colonoscopy and pathology results are completed. The nature of communication is at the providers’ discretion, including in-person visits, telephone calls, messages through patient portals, or mailed letters. After the initial recommendation is conveyed, there is no standardized system for further reminders about the need for surveillance and any subsequent communication occurs at the providers’ discretion. Similarly, such communication typically occurs either in-person during a clinic visit or remotely through 3 modalities (telephone call, electronic message through an online patient portal, or mailed letters).

As part of our efforts to improve CRC outcomes in our institution, we aimed to determine the magnitude of missed surveillance among patients with adenomas with high-risk features and to identify modifiable predictors of surveillance uptake as potential targets for future interventions to improve surveillance rates. We hypothesized that timely surveillance would be low and that patient contact with the health system (e.g., provider visits) and documentation of adenoma diagnosis would be associated with increased likelihood of surveillance uptake.

## Methods

University of California Los Angeles (UCLA) Health is a large, integrated, tertiary care academic medical center in Southern California with a defined primary care population. The primary care population includes over 371,000 enrollees, each assigned to one primary care physician (PCP) who provides referrals to specialty care services such as gastroenterology (GI) when indicated.

**Figure 1:**
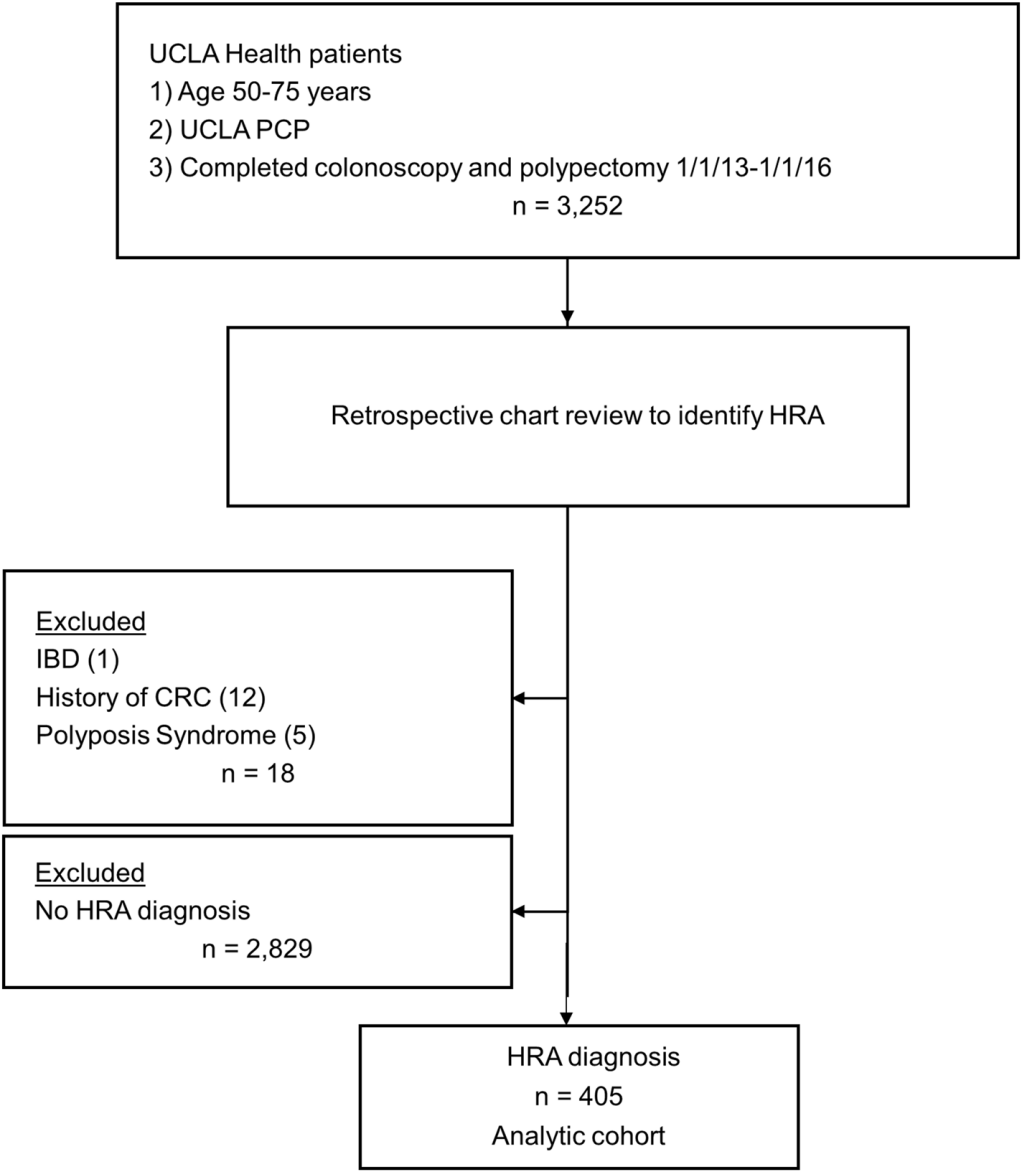
Flowchart of patient exclusions to obtain final analytic cohort.

We performed an automated query of the electronic health record (EHR) to determine patients (1) aged 50–75; (2) assigned to a UCLA PCP (i.e., regular primary care patient); (3) who underwent index colonoscopy with polypectomy between January 1, 2013 and January 1, 2016. The resulting 3252 patients (Fig. 1) were randomized and assigned to one person on a team of 9 abstractors (one gastroenterologist, one general internist, one gastroenterology quality improvement fellow, four 2nd year internal medicine residents, two 3rd year medical students) trained on a standardized abstraction protocol who manually reviewed patient charts until at least 400 patients with an adenoma with high-risk features (as defined by the following criteria: 1 or more TA ≥ 1 cm, TA with tubulovillous or villous histology, TA with high-grade dysplasia, or 3 or more TA of any size) were identified. As our intervention was intended for quality improvement purposes, we did not perform an a priori sample size calculation. However, we were powered to detect a 15% difference in surveillance utilization, assuming a baseline rate of 45%, two-arm randomization, 80% power and an alpha level of 0.05. Chart abstractions were reviewed in real-time by the lead investigators, any identified discrepancies were fixed, and feedback was provided to abstractors to ensure a final interrater reliability > 90%. We relied on the definition of HRA used by the 2012 MSTF surveillance guidelines which were contemporaneous to the study period^6^. Sessile serrated adenomas (SSA) were not included in those HRA diagnostic criteria. Patients with a history of CRC, Crohn’s disease, ulcerative colitis, or polyposis syndrome were excluded given these conditions influence recommended surveillance intervals. All abstractors underwent standardized training using a common training set of cases to ensure a high degree of interrater agreement (> 90%) and to correct any deviations from protocol. In addition, all abstractions were reviewed by one team member throughout the data collection process.

The primary outcome was uptake of surveillance colonoscopy within 3.5 years of index colonoscopy. This timeframe was based on the 2012 MSTF recommended surveillance interval of 3 years for patients diagnosed with HRA, with an additional 0.5-year grace period recognizing that colonoscopy often demands time off work, a personal escort, and other arrangements that may require extra time^6^. Time to surveillance colonoscopy was calculated as the duration of time between the index colonoscopy and surveillance completion. Secondary outcomes included cumulative rates of surveillance colonoscopy uptake at 0.5-year intervals after index colonoscopy.

To identify barriers and facilitators of surveillance uptake, abstractors collected data on patient demographics (age, gender, race/ethnicity), clinical factors (family history, presence of adenoma on the EHR problem list, endoscopic and histologic characteristics of HRAs identified on index colonoscopy), PCP and GI visitation following index colonoscopy (number, time from index colonoscopy), provider use of patient surveillance reminders (mail, telephone, or electronic patient portal messages at the provider’s discretion), and specialty of the surveillance colonoscopy ordering provider (PCP, GI, other). Surveillance colonoscopy ordering provider is defined as the provider who personally ordered the surveillance colonoscopy in the EHR. The UCLA Clinical and Translational Science Institute (CTSI) provided data for insurance status at time of index and surveillance colonoscopies and comorbidity, which we merged with the manually abstracted dataset. We summarized comorbidity with the Charlson Comorbidity Index (CCI), a weighted scoring system that integrates International Classification of Disease (ICD) 9 and 10 data on 17 clinical features to predict survival and for which increasing values represent increasing comorbidity^12^. Insurance groupings were mutually exclusive based on the following hierarchy of insurance types with the former taking precedence over the latter: UCLA managed care, commercial insurance, Medicare, Medi-Cal/Medicaid, and other insurance type. For race and ethnicity, we used six mutually exclusive categories: Non-Hispanic white, Hispanic, Non-Hispanic Black, Non-Hispanic Asian and Pacific Islander, Non-Hispanic other, and unknown. All patients were followed until surveillance colonoscopy completion or for at least 4 years.

We calculated means (SD) for continuous variables and frequencies (%) for categorical variables to describe the patient cohort overall and by colonoscopy completion status at 3.5 years. We used Student’s *t* tests and χ^2^ tests to investigate differences in baseline patient characteristics between those who completed surveillance colonoscopy at 3.5 years and those who did not. We then determined the proportion of patients who completed surveillance at each 0.5-year interval. Among those who completed surveillance, we also calculated median time (with interquartile range) to colonoscopy.

We used univariate and multivariable logistic regression models to identify significant predictors of surveillance completion. Demographic factors were age, gender, and race/ethnicity. Clinical factors were CCI, family history, presence of adenoma on the EHR problem list, and endoscopic and histologic characteristics of HRAs identified on index colonoscopy. Health system and service utilization factors included insurance status when surveillance was due, PCP and GI visitation following index colonoscopy, provider use of patient surveillance reminders, and specialty of the ordering provider of surveillance colonoscopy. The final multivariable model included demographic variables and variables significantly associated with the primary outcome in univariate analysis (*P* values < 0.05). All remaining variables had less than 2% missing values. All analyses were conducted using SAS, version 9.4 (SAS Institute, Inc.; Cary, NC, USA). The study was reviewed and approved by the UCLA Institutional Review Board, and informed consent was waived due to minimal risk research. All methods were carried out in accordance with institutional guidelines and regulations.

## Results

### Study population and descriptive characteristics

Figure 1 summarizes the exclusions that yielded our final analytic cohort of 405 patients. Mean age was 62.2 (7.4) years, 63.0% were male, and 68.4% were Non-Hispanic White (Table 1). Mean CCI was 3.2 (3.3). In all, 56% of patients had a diagnosis of adenoma listed on the EHR problem list, 15.6% had a family history of CRC, and 95.2% were insured. The maximum follow-up duration was 6.6 years (median 3.9 years; interquartile range 3.0–4.6).

**Table 1:** Demographic and clinical characteristics for the total patient cohort, overall and by surveillance status at 3.5 years.

	Total n = 405	Surveillance incomplete at 3.5 years n = 233	Surveillance complete at 3.5 years n = 172	*P* value
	n (%)	n (%)	n (%)	
Mean Age ± SD	62.2 ± 7.4	62.2 ± 7.5	62.1 ± 7.4	0.90
**Gender**				
Male gender	255 (63.0)	149 (63.9)	106 (61.6)	0.63
Female gender	150 (37.0)	84 (36.1)	66 (38.4)	
**Race/ethnicity**				
Non-hispanic white	277 (68.4)	162 (69.5)	115 (66.9)	0.80
Hispanic	37 (9.1)	23 (9.9)	14 (8.1)	
Non-hispanic black	37 (9.1)	19 (8.2)	18 (10.5)	
Non-hispanic Asian/Pacific Islander	30 (7.4)	16 (6.9)	14 (8.1)	
Non-hispanic other race	18 (4.4)	9 (3.9)	9 (5.2)	
Unknown	6 (1.5)	4 (1.7)	2 (1.2)	
Mean CCI ± SD	3.2 ± 3.3	3.2 ± 3.6	3.1 ± 3.0	0.78
**Adenoma on EHR problem list**				
Yes	227 (56.0)	124 (53.2)	103 (59.9)	0.19
No	178 (44.0)	109 (46.8)	69 (40.1)	
**Family history of CRC**				
Yes	63 (15.6)	37 (15.9)	26 (15.1)	0.83
No	342 (84.4)	196 (84.1)	146 (84.9)	
**Any surveillance reminder**				
Yes	238 (58.8)	122 (52.4)	116 (67.4)	< 0.01*
No	167 (41.2)	111 (47.6)	56 (32.6)	
**Type of reminder**^a^				
Patient email only	18 (7.6)	8 (6.6)	10 (8.6)	0.11
Patient letter only	151 (63.4)	86 (70.5)	65 (56.0)	
Telephone call only	51 (21.4)	22 (18.0)	29 (25.0)	
Multiple reminder types	18 (7.6)	6 (4.9)	12 (10.3)	
**Colonoscopy ordering provider**				
PCP	196 (48.4)	84 (36.1)	112 (65.1)	< 0.01*
GI	80 (19.8)	21 (9.0)	59 (34.3)	
Other	1 (0.2)	1 (0.4)	0 (0)	
Unknown specialty	3 (0.7)	2 (0.9)	1 (0.6)	
No order	125 (30.9)	125 (53.6)	0 (0)	
**PCP visits within 3 years after index**				
0–5	149 (36.8)	86 (36.9)	63 (36.6)	0.56
6–10	124 (30.6)	67 (28.8)	57 (33.1)	
Above 10	132 (32.6)	80 (34.3)	52 (30.2)	
**GI visit within 3 years after index**				
0 visits	306 (75.6)	192 (82.4)	114 (66.3)	< 0.01*
1–2	77 (19.0)	30 (12.9)	47 (27.3)	
Above 2	22 (5.4)	11 (4.7)	11 (6.4)	
**Insurance status (at surveillance due date)**^b^				
No insurance	19 (4.7)	14 (6.0)	5 (2.9)	0.40
UCLA managed care	122 (30.1)	66 (28.3)	56 (32.6)	
Commercial	214 (52.8)	122 (52.4)	92 (53.5)	
Medicare/Medical/Other	50 (12.3)	31 (13.3)	19 (11.1)	
**HRA subtype**^c^				
TA ≥ 1 cm	163 (40.2)	95 (40.8)	68 (39.5)	0.80
High grade dysplasia	16 (4.0)	6 (2.6)	10 (5.8)	0.10
Tubulovillous/Villous histology	121 (29.9)	63 (27.0)	58 (33.7)	0.15
≥ 3 TA	194 (47.9)	102 (43.8)	92 (53.5)	0.05

*CCI* Charlson Comorbidity Index; *CRC* colorectal cancer; *EHR* electronic health record; *GI* gastroenterologist; *HRA* high risk adenoma; *PCP* primary care physician; *SD* standard deviation; *TA* tubular adenoma.

^a^Comparing only among those receiving a reminder; n = 238.

^b^Insurance groupings are mutually exclusive.

^c^HRA subtypes are not mutually exclusive.

*Denotes statistical significance at the *P* < 0.05 level.

% are column percents unless otherwise indicated.

### Surveillance uptake

**Figure 2:**
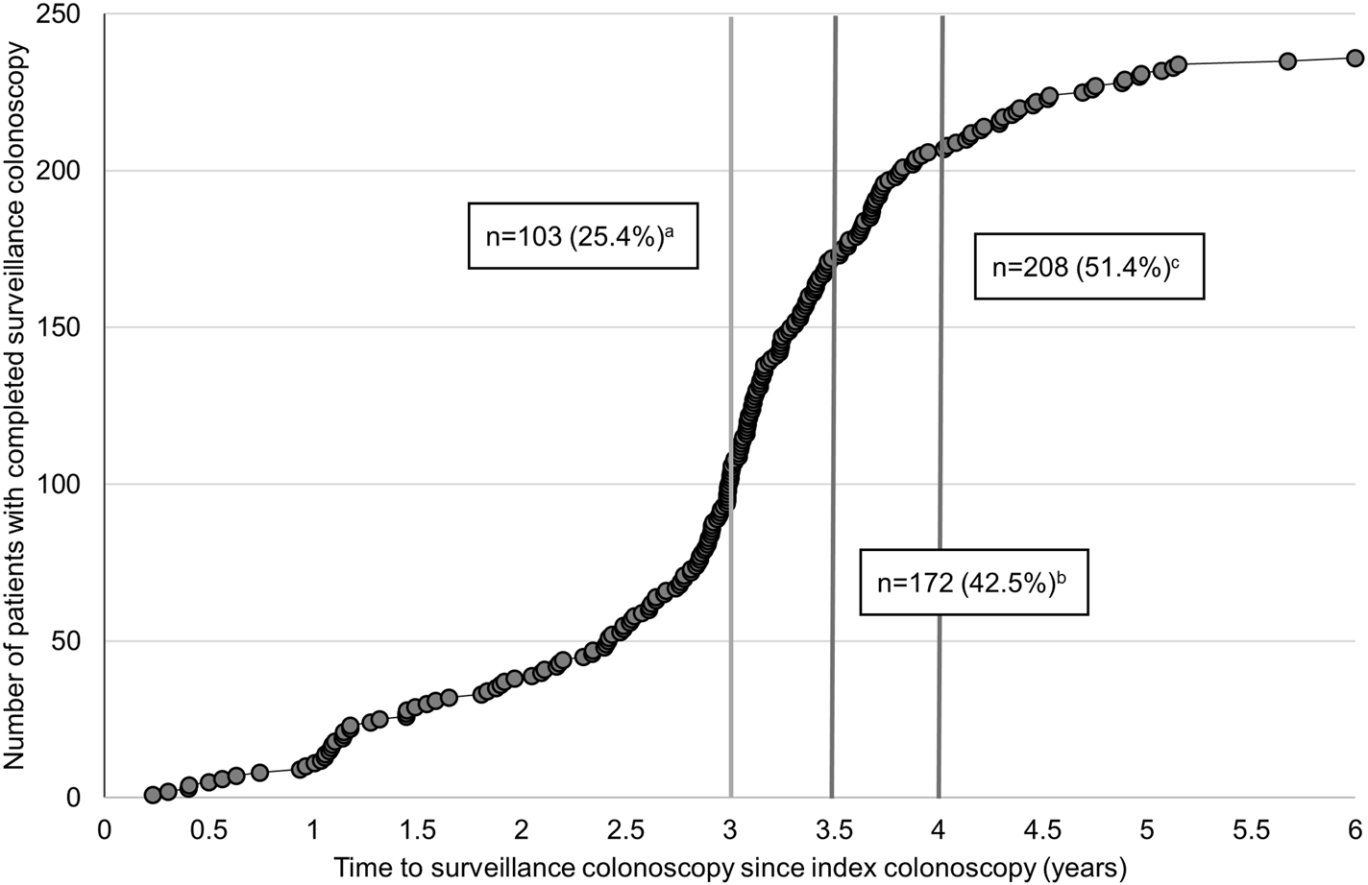
Cumulative frequency of patients with completed surveillance over time; n = 405. a, b, and c represent the cumulative frequency (% of total cohort) of patients completing uptake by 3, 3.5, and 4 years, respectively. Data points at 4.5, 5, 5.5, and 6 years only include patients with follow-up at least that duration.

Overall, surveillance uptake was low (Fig. 2). At 3.5 years after index colonoscopy, 172/405 (42.5%) of patients with HRA had completed surveillance colonoscopy. The surveillance rate was 103/405 (25.4%) at 3 years and 208/405 (51.4%) at 4 years. Of note, colonoscopy participation increased dramatically (17.1%) between 3 and 3.5 years, followed by a continued but slower rise (8.9%) in uptake between 3.5 and 4 years. Among those who completed surveillance colonoscopy, median time to surveillance was 3.1 years (interquartile range 2.6–3.6).

### Predictors of surveillance completion

In bivariate analysis, there were several differences between those who completed surveillance at 3.5 years and those who did not (Table 1). The occurrence of any reminder was significantly higher among those who completed surveillance colonoscopy at 3.5 years compared to those who did not (67.4% v. 52.4%; *P* < 0.01). The prevalence of having 1 or more GI visits in the 3 years following HRA diagnosis was significantly higher among those who completed surveillance compared to those who did not (*P* < 0.01). Among those who had a surveillance colonoscopy ordered, having a GI provider order the colonoscopy was associated with a higher rate of surveillance completion compared to having a PCP order the colonoscopy (59/80 or 73.8% v. 112/196 or 57.1%; *P* < 0.01). We did not observe a significant difference in the prevalence of family history or in HRA subtypes between those who completed surveillance and those who did not (Table 1).

**Figure 3:**
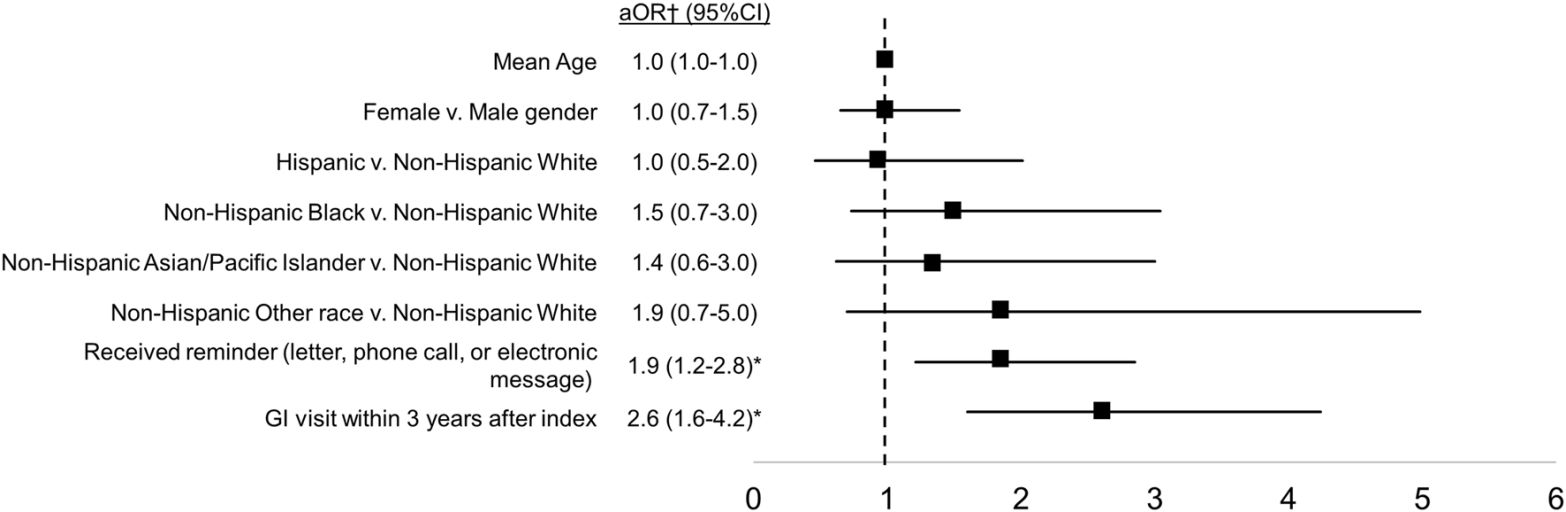
Predictors of surveillance colonoscopy completion at 3.5 years; n = 405. †For the multivariable model, we included age, gender, race/ethnicity, and variables significant at the *P* < 0.05 level in univariate analyses. Ordering provider was only relevant for patients who had a colonoscopy order and was therefore excluded. *Denotes statistical significance at the *P* < 0.05 level. *aOR* adjusted odds ratio; *CI* confidence interval; *GI* gastroenterology.

When controlling for relevant confounders, we observed two significant predictors of surveillance uptake at 3.5 years after HRA diagnosis (Fig. 3 and Table 2). The odds of completing surveillance increased significantly for patients who received a reminder (any type) that surveillance was due (adjusted OR = 1.9; 95% CI = 1.2–2.8) and for patients who had at least one GI clinic visit in the 3 years following an HRA diagnosis (adjusted OR = 2.6; 95% CI = 1.6–4.2). In contrast, visitation with PCP during the same time period did not predict surveillance completion.

**Table 2:** Crude and adjusted odds ratios of surveillance colonoscopy completion at 3.5 years; n = 405.

	Unadjusted OR (95%CI)	Adjusted OR^a^ (95%CI)
Age	1.0 (1.0–1.0)	1.0 (1.0–1.0)
Female versus male gender	1.1 (0.7–1.7)	1.0 (0.7–1.5)
**Race/ethnicity**		
Hispanic v. Non-Hispanic White	0.9 (0.4–1.7)	1.0 (0.5–2.0)
Non-Hispanic Black v. Non-Hispanic White	1.3 (0.7–2.7)	1.5 (0.7–3.0)
Non-Hispanic Asian/Pacific Islander v. Non-Hispanic White	1.2 (0.6–2.6)	1.4 (0.6–3.0)
Non-Hispanic Other race v. Non-Hispanic White	1.4 (0.5–3.7)	1.9 (0.7–5.0)
CCI	1.0 (1.0–1.1)	
Adenoma on EHR Problem List	1.3 (0.9–2.0)	
Family History of CRC v. no family history	1.0 (0.6–1.6)	
Received any reminder v. no reminder	1.9 (1.2–2.8)*	1.9 (1.2–2.8)*
Colonoscopy Ordering Provider GI v. PCP^b^	1.9 (1.0–3.4)*	
**PCP visits within 3 years after index**		
6–10 v. 0–5	0.9 (0.6–1.5)	
≥ 11 v. 0–5	1.2 (0.7–1.9)	
GI visit within 3 years after index v. no visit	2.4 (1.5–3.8)*	2.6 (1.6–4.2)*
**Insurance Status (at surveillance due date)**^c^		
Commercial v. UCLA Managed Care	0.9 (0.6–1.4)	
Medicare/Medical/Other v. UCLA Managed Care	0.7 (0.4–1.4)	
**HRA subtype**^d^		
TA ≥ 1 cm v. no TA ≥ 1 cm	1.0 (0.6–1.4)	
High-grade dysplasia v. no high-grade dysplasia	2.3 (0.8–6.6)	
Tubulovillous/Villous histology v. no Tubulovillous/Villous histology	1.4 (0.9–2.1)	
≥ 3 TA v. < 3 TA	1.5 (1.0–2.2)	

*CI* confidence interval; *CCI* Charlson Comorbidity Index; *EHR* electronic health record; *CRC* colorectal cancer; *GI* gastroenterologist; *OR* odds ratio; *PCP* primary care physician; *HRA* high risk adenoma; *TA* tubular adenoma.

^a^For adjusted models, we included age, gender, race/ethnicity, and variables significant at the *P* < 0.05 level in univariate analyses.

^b^Total n = 280 patients with a colonoscopy order. Because only 69.1% of patients had a colonoscopy order, this variable was not included in the multivariable logistic regression.

^c^Among patients with insurance n = 386.

^d^HRA subtypes are not mutually exclusive.

*Denotes statistical significance at the *P* < 0.05 level.

## Discussion

We found that uptake of surveillance colonoscopy often occurred after the 3-year surveillance interval and remained low 4 years after HRA diagnosis in a large and integrated academic health network. In adjusted analysis, we also demonstrated that patients who saw GI in consultation at least once or who received an electronic, telephone, or mailed reminder that surveillance was due were more likely to complete surveillance in a timely manner. These findings indicate that short-interval surveillance practice patterns require increased attention to combat the ongoing CRC burden and that there are factors that can serve as focused, intervenable targets to assist in this endeavor. In the setting of new post-polypectomy surveillance guidelines that include more indications for the 3-year surveillance interval, our findings underscore the need for a proactive approach to achieve surveillance goals.

Our findings are consistent with a pattern of low surveillance uptake observed in other clinical settings. In a retrospective cohort study exploring surveillance patterns in community practice, Schoen et al. found low rates of surveillance uptake at 3 years for patients diagnosed with an aTA (30.7%) or ≥ 3 non-advanced adenomas (19.5%)^8^. Across several large integrated health systems, Chubak et al. reported similarly low uptake at 3 years for patients diagnosed with ≥ 3 adenomas and/or ≥ 1adenoma with villous/tubulovillous histology (10.2–30.9%)^9^. Akin to our findings, both studies noted a sudden rise in surveillance uptake in the immediate wake of the surveillance due date, and subsequent studies have shown increases in surveillance colonoscopy up to 6 years after HRA diagnosis, well after the recommended 3-year interval^8,11,13^. Though early surveillance was not a focus of this study, we note that there were 25% of patients who underwent colonoscopy before the 3-year due date, which likely reflects diagnostic colonoscopy for interval symptom development (e.g. rectal bleeding) in addition to early surveillance.

The low rates of surveillance uptake observed in the literature and in our own study illustrate the ongoing issue of untimely, inadequate surveillance for patients with adenomas with high-risk features and highlight the importance of targeting factors that will maximize timely surveillance uptake. Based on our institutional experience, most providers communicate the need for surveillance colonoscopy to patients after the index colonoscopy through a variety of methods (i.e. telephone call, electronic patient portal, mailed letter, follow-up visit), but there is no standardized system for subsequent communication or reminders and any future communication occurs at the providers’ discretion or on an opportunistic basis (e.g. at a follow-up visit for unrelated reasons during which a routine GI-focused interview reveals a history of high risk polyps). While several predictors of surveillance uptake such as age and family history of CRC in a first-degree relative have been identified in the literature^8,9,11,14^, these factors are difficult to translate into interventions that can readily improve surveillance uptake. One study by Braschi et al. found that PCP visitation and documentation of index pathology findings on the EHR problem list were both associated with surveillance uptake^14^. We expand upon the existing literature through the identification of two new modifiable factors predictive of surveillance colonoscopy completion at 3.5 years that provide potential avenues for system-level interventions.

In particular, patient reminders present a broadly accessible and high-yield target for future work. In the CRC screening literature, there is precedent for patient reminders as a powerful tactic for improving screening uptake of average-risk patients^15–17^. Reminders could have a similarly positive impact on surveillance uptake. Moreover, our finding that reminder modality (whether electronic patient portal, mailed letter, or telephone call) had no association with surveillance completion implies that many types of reminders that are accessible within a health system may possess similar potential to improve chances of surveillance uptake. In short, any reminder is likely better than none. Lastly, patient reminders have been found to be a cost effective tool for improving CRC screening uptake, thus broadening their appeal to a variety of healthcare settings^18^.

Our study also uniquely identified consultation with GI as a significant predictor of surveillance uptake. The specific aspects of GI consultation that improve surveillance uptake are not immediately clear, but there are several conceivable explanations for this observation. First, while our health system has a robust primary care network, it is also a tertiary care center where the care of patients is often specialist-driven. GIs may be able to focus on specialty-specific issues in more detail and have more familiarity with surveillance guidelines than PCPs. Secondly, GIs may have more experience conveying to patients the implications of an adenoma with high-risk features, addressing patient questions about periprocedural and procedural risks/benefits, and directly coordinating with endoscopy scheduling teams to facilitate surveillance completion. Third, given the procedure-focused reimbursement structure, gastroenterologists may be more incentivized to proactively pursue surveillance colonoscopy. Lastly, patients with GI-related symptoms that would not in themselves warrant colonoscopy (e.g. acid-reflux or dyspepsia) may prompt more frequent GI visitation during which a routine GI-focused history may reveal need for surveillance colonoscopy.

Although it is likely not practical or feasible to schedule a GI provider visit with the sole intention to capture patients due for surveillance, our findings suggest there may be aspects of the GI visit that lead to higher surveillance uptake and could be captured in a more cost/time-effective strategy, such as the utilization of patient navigators to help patients overcome educational and logistic barriers to surveillance completion. Indeed, patient navigation has demonstrated efficacy in the CRC screening literature^19–21^. Additionally, implementation of an automated recall system would provide for an additional safety-net to make sure the need for surveillance is not forgotten several years after the index diagnosis.

While PCP visitation was not significantly associated with surveillance uptake in our study, our inclusion criteria required established care with a UCLA PCP which may have mitigated any PCP-related impacts on uptake. Moreover, there may be institutional differences in departmental ownership of surveillance follow-up. Our combined findings bring to light one compelling explanation for persistently low uptake: the need for surveillance colonoscopy in eligible patients is often simply forgotten. This idea is supported by three observations: (1) patient reminders and provider visits were strongly associated with timely surveillance; (2) there was a rapid uptake in surveillance just after the due date, which suggests patients, providers, or system-level reminders are helping to recognize when surveillance is overdue; and (3) we, alongside many other studies, did not find an association between increased comorbidity and lack of surveillance uptake, which suggests that there was not a compelling clinical reason for many patients to defer surveillance other than both providers and patients forgetting to complete surveillance. These findings further emphasize the importance of reminder-based interventions.

Our study had several limitations. First, we used a single integrated health system with a highly insured population, which may limit the generalizability of our findings. However, our findings are relevant to large integrated academic health systems that represent millions of patients and inform quality of care in these settings. Second, we may have missed surveillance colonoscopies performed outside our health system. However, this possibility is minimized due to (1) the efforts of a team of patient navigators from the population health department who are dedicated to obtaining and uploading outside colonoscopy reports for primary care enrollees, (2) our abstraction team’s review of all outside procedures scanned into the EHR, and (3) limiting the cohort to patients within the UCLA primary care network who are more likely to obtain in-network colonoscopies. Third, we did not account for participation in colonoscopy before the index colonoscopy, and patients with previous abnormal colonoscopies may have higher rates of subsequent colonoscopy independent of an HRA diagnosis. Lastly, new MSTF surveillance guidelines have been released since the completion of this work which include changes in the criteria for high-risk features and associated surveillance intervals. However, the findings in this study are still relevant because they demonstrate poor adherence to de facto surveillance guidelines and highlight modifiable predictors of compliance with surveillance guidelines which will assist institutions as they implement the new surveillance guidelines.

Despite the above limitations, our study has several strengths. First, our focus on adenomas with high-risk features sheds light on a less-studied aspect of the CRC care continuum and CRC prevention strategies. Secondly, in contrast to many prior studies which only focused on a subset of HRA criteria, we were able to reliably abstract data on all HRA subtypes, producing a cohort that matched the HRA definition used by the 2012 MSTF polyp surveillance guidelines. Third, we employed a rigorous methodology for our manual chart abstraction to generate a high-quality database, which contributes to the validity and reliability of the study findings. Finally, our study contributes a retrospective analysis from a large integrated healthcare network with multivariable logistic regression to identify modifiable predictors of surveillance uptake that serve as potential targets for future interventions. Both of the significant predictors of surveillance that we identified (GI consultation and patient reminders) are broadly applicable in most health systems.

In conclusion, our findings highlight the ongoing issue of low uptake of short-interval colonoscopic surveillance and provide potential targets for future health system interventions through the identification of modifiable factors associated with increased surveillance uptake. Future efforts should implement and evaluate the impact of system-level patient reminders and other strategies to move us towards evidence-based interventions that increase timely surveillance uptake. Successful new approaches can be tailored for health systems challenged with identifying and recalling patients at elevated risk for CRC and will improve health outcomes for one of the most common and deadly malignancies in the US.

## Data Availability

Raw data were generated at UCLA Health. Derived data supporting the findings of this study are available from the corresponding author Folasade P. May (FPM) on request.

## References

[1] Howlader, N. *et al.* SEER Cancer Statistics Review, 1975–2016, National Cancer Institute.

[2] Lin, J. S. *et al. Screening for Colorectal Cancer: A Systematic Review for the U.S. Preventive Services Task Force*. (Agency for Healthcare Research and Quality (US), 2016).

[3] Zauber AG (2012). Colonoscopic polypectomy and long-term prevention of colorectal-cancer deaths. N. Engl. J. Med..

[4] Mandel JS (2000). The effect of fecal occult-blood screening on the incidence of colorectal cancer. N. Engl. J. Med..

[5] Winawer SJ (1993). Prevention of colorectal cancer by colonoscopic polypectomy. The National Polyp Study Workgroup. N. Engl. J. Med..

[6] Lieberman DA (2012). Guidelines for colonoscopy surveillance after screening and polypectomy: A consensus update by the US Multi-Society Task Force on Colorectal Cancer. Gastroenterology.

[7] Gupta S (2020). Recommendations for follow-up after colonoscopy and polypectomy: A consensus update by the US Multi-Society Task Force on Colorectal Cancer. Am. J. Gastroenterol..

[8] Schoen RE (2010). Utilization of surveillance colonoscopy in community practice. Gastroenterology.

[9] Chubak J (2018). Receipt of colonoscopy following diagnosis of advanced adenomas: An analysis within integrated healthcare delivery systems. Cancer Epidemiol. Prev. Biomark..

[10] Green BB (2018). Colorectal cancer control: Where have we been and where should we go next?. JAMA Intern. Med..

[11] Laiyemo AO (2009). Utilization and yield of surveillance colonoscopy in the continued follow-up study of the polyp prevention trial. Clin. Gastroenterol. Hepatol. Off. Clin. Pract. J. Am. Gastroenterol. Assoc..

[12] Quan H (2005). Coding algorithms for defining comorbidities in ICD-9-CM and ICD-10 administrative data. Med. Care.

[13] Murphy CC, Sandler RS, Grubber JM, Johnson MR, Fisher DA (2016). Underuse and overuse of colonoscopy for repeat screening and surveillance in the veterans health administration. Clin. Gastroenterol. Hepatol. Off. Clin. Pract. J. Am. Gastroenterol. Assoc..

[14] Braschi C (2014). Patient-, provider-, and system-level factors in low adherence to surveillance colonoscopy guidelines: Implications for future interventions. J. Gastrointest. Cancer.

[15] Inadomi JM (2012). Adherence to colorectal cancer screening: a randomized clinical trial of competing strategies. Arch. Intern. Med..

[16] Sequist TD, Zaslavsky AM, Marshall R, Fletcher RH, Ayanian JZ (2009). Patient and physician reminders to promote colorectal cancer screening: a randomized controlled trial. Arch. Intern. Med..

[17] Green BB (2013). An automated intervention with stepped increases in support to increase uptake of colorectal cancer screening: a randomized trial. Ann. Intern. Med..

[18] Sequist TD, Franz C, Ayanian JZ (2010). Cost-effectiveness of patient mailings to promote colorectal cancer screening. Med. Care.

[19] Percac-Lima S (2009). A culturally tailored navigator program for colorectal cancer screening in a community health center: a randomized, controlled trial. J. Gen. Intern. Med..

[20] Lasser KE (2009). A multilevel intervention to promote colorectal cancer screening among community health center patients: results of a pilot study. BMC Fam. Pract..

[21] Christie J (2008). A randomized controlled trial using patient navigation to increase colonoscopy screening among low-income minorities. J. Natl. Med. Assoc..

